# The voyage is as important as the harbor

**DOI:** 10.7554/eLife.96836

**Published:** 2024-03-15

**Authors:** Anton Sabantsev, Sebastian Deindl

**Affiliations:** 1 Department of Cell and Molecular Biology, Uppsala University Uppsala Sweden

**Keywords:** chromatin remodelling, nucleosome sliding, DNA, nucleosome searching, transcription, 1D diffusion, *S. cerevisiae*

## Abstract

To find nucleosomes, chromatin remodelers slide and hop along DNA, and their direction of approach affects the direction that nucleosomes slide in.

**Related research article** Kim JM, Carcamo CC, Jazani S, Xie Z, Feng XA, Yamadi M, Poyton M, Holland KL, Grimm JB, Lavis LD, Ha T, Wu C. 2023. Dynamic 1D search and processive nucleosome translocations by RSC and ISW2 chromatin remodelers. *eLife* **12**:RP91433. doi: 10.7554/eLife.91433.

Most of the DNA in eukaryotic cells is wrapped around histone proteins in a spool-like configuration to form basic structural units called nucleosomes, which together make up chromatin (Luger et al., 1997; Kornberg, 1974). Such tight packaging significantly restricts DNA accessibility (Kornberg and Lorch, 2020). As a result, the position of nucleosomes around a gene promoter – the stretch of DNA where gene expression is initiated – can dictate whether a gene is turned on or off (Jiang and Pugh, 2009).

Chromatin remodelers are proteins powered by ATP that play a crucial role in sliding nucleosomes along DNA to ensure specific genomic sequences are accessible. Some chromatin remodelers, like those known as RSC and ISW2 in yeast, operate at promoters by either pushing nucleosomes apart (RSC) or pulling them together (ISW2) (Yen et al., 2012). This increases or decreases the accessibility of the promotor, respectively. Understanding how the chromatin remodelers exert these opposing effects is essential for comprehending the organization of a genome and the pathogenesis of many diseases with genetic components – including cancers.

In principle, a protein can find its target simply by randomly diffusing in all three spatial dimensions. However, navigating the vast genomic landscape using only these random 3D movements would be an exceedingly time-consuming endeavor. Therefore, many DNA-binding proteins have evolved to bind weakly to any DNA sequence and move along it, a process known as 1D diffusion. This combination of 3D and 1D diffusion greatly speeds up the search process (Berg et al., 1981; Marklund et al., 2020). Now, in eLife, Carl Wu, Taekjip Ha and colleagues – including Jee Min Kim and Claudia Carcamo as joint first authors – report that the yeast chromatin remodelers RSC and ISW2 diffuse over long stretches of DNA to find nucleosomes to slide. Moreover, their direction of approach affects the direction that nucleosomes slide in. Taken together, these findings reveal an intriguing new facet of remodeler function (Kim et al., 2023).

The team (who are based at Johns Hopkins University and HHMI Janelia Research Campus) used optical tweezers to hold stretches of DNA in place (van Mameren et al., 2009; Comstock et al., 2011). Combined with confocal fluorescence microscopy techniques, this allowed them to visualize individual fluorescently labeled molecules of RSC and ISW2 diffusing along DNA molecules with widely spaced nucleosomes (Figure 1A). The experiments demonstrated that both remodelers can diffuse along DNA, but that each displays distinct diffusion mechanisms. ISW2 slides along the DNA, following its spiral configuration closely, whereas RSC hops between nearby positions on the DNA strand (see Figure 1B). Additionally, labeling remodelers with different fluorescent markers allowed simultaneous tracking of their movements on the same DNA molecule. Interestingly, when two remodelers encounter each other while diffusing along DNA, they seldom pass by each other. Instead, they either recoil or briefly come together to co-diffuse for less than one second.

**Figure 1.**
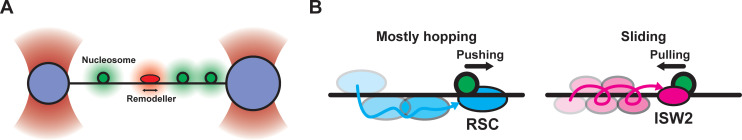
How remodelers find nucleosomes. (**A**) Schematic of the assay used to observe chromatin remodeler 1D diffusion and subsequent nucleosome sliding on a sparse nucleosome array. A long DNA molecule (approximately 50,000 base pairs) with up to 40 nucleosomes is stretched between two microspheres held in optical traps (purple). Fluorescence from nucleosomes (green) and chromatin remodeler molecules (red) is monitored using confocal fluorescence microscopy. (**B**) Schematics illustrating the target search mechanisms and resulting nucleosome sliding directionality for RSC (left) and ISW2 (right) remodelers.

When remodelers arrive at a nucleosome, they often attach to it and then begin remodeling. In the presence of ATP, Kim et al. noted that RSC or ISW2 remodelers bind to nucleosomes and then slide them in a single direction for considerable distances, sometimes exceeding 500 base pairs. This behavior distinguishes them from remodelers such as Chd1, which frequently alter their direction (Qiu et al., 2017). The capability to visualize individual remodelers locating and subsequently moving a nucleosome in real-time also revealed a fascinating and unexpected effect – the direction of nucleosome sliding depends on the direction the remodeler approaches the nucleosome from. RSC nudges nucleosomes onward in the same direction as it was moving before, while ISW2 pulls them in the opposite direction from which it arrived (Figure 1B).

Until now, real-time single-molecule analysis of nucleosome sliding was mainly achieved using single-molecule FRET – a potent method capable of detecting movements of a couple of base pairs, but with a limited dynamic range of less than 20 base pairs. As a result, very little was known about the behavior of chromatin remodelers at larger scales. The findings of Kim et al. suggest an intriguing new mechanism behind the effects of RSC and ISW2 on promoter nucleosomes: the stretches of nucleosome-free DNA near promoters serve as landing sites for these remodelers, allowing them to use 1D diffusion to move towards the nucleosomes. This might not only help remodelers locate the nucleosomes they need to act upon, but also orient them to slide nucleosomes in the right direction.

In the future, it would be fascinating to see the techniques used by Kim et al. extended to other remodelers, including those found in humans, to examine how widespread the observed effects are. Furthermore, this approach is well-positioned to explore the critical question of what occurs when a remodeler moves a nucleosome into another nucleosome or obstacle. Developing a quantitative model that integrates 1D diffusion could facilitate analysis of the impact of these previously unappreciated effects on sliding of promoter nucleosomes. Constructing such a model necessitates further characterization of nucleosome binding rates via both 1D and 3D diffusion. Overall, these important findings will undoubtedly shape future research and encourage scientists to consider how remodelers locate nucleosomes in their experiments and the potential consequences for the remodeling process.
